# Changes in Physical Inactivity Among US Adults Overall and by Sociodemographic Characteristics, Behavioral Risk Factor Surveillance System, 2020 Versus 2018

**DOI:** 10.5888/pcd20.230012

**Published:** 2023-07-27

**Authors:** Miriam E. Van Dyke, Tiffany J. Chen, Jasmine Y. Nakayama, Latetia V. Moore, Geoffrey P. Whitfield

**Affiliations:** 1 Physical Activity and Health Branch, Division of Nutrition, Physical Activity, and Obesity, Centers for Disease Control and Prevention, Atlanta, Georgia; 2 McKing Consulting Corporation, Atlanta, Georgia; 3 Office of Science, Division of Nutrition, Physical Activity, and Obesity, Centers for Disease Control and Prevention, Atlanta, Georgia

## Abstract

The COVID-19 pandemic may have disrupted people’s work–life patterns and access to places to be physically active. Behavioral Risk Factor Surveillance System data were analyzed to assess changes in self-reported leisure-time physical inactivity. The results showed that prevalence of inactivity among US adults decreased 0.7 percentage points (95% CI: −1.2 to −0.3), from 24.5% in 2018 to 23.8% in 2020, and the greatest decreases were observed among rural-dwelling women, rural-dwelling men, and non-Hispanic White women. These findings highlight a need to understand and address factors that lead to differential changes in leisure-time physical inactivity across subpopulations during public health emergencies.

**Summary**

**What is already known on this topic?**

Studies of physical activity changes during 2020, the first year of the COVID-19 pandemic, have produced mixed findings.

**What is added by this report?**

National data among US adults from the Behavioral Risk Factor Surveillance System suggest that leisure-time physical inactivity modestly decreased overall and among specific US subpopulations during 2020 versus 2018. Decreases occurred primarily among women, most notably among rural-dwelling women and non-Hispanic White women. Rural-dwelling men also reported modest decreases.

**What are the implications for public health practice?**

This study highlights a need to understand and address factors that may lead to differential changes in leisure-time physical inactivity across subpopulations during public health emergencies.

## Objective

Physical activity has many health benefits, including reducing anxiety, improving sleep, and lowering blood pressure, as well as lowering the risk of type 2 diabetes, heart disease, and some cancers (1). Physical activity also helps prevent severe outcomes from COVID-19 (2), which the World Health Organization declared a pandemic in March 2020.

Early in the pandemic, uneven access to safe places for physical activity and shifting work–life demands may have exacerbated existing disparities in physical activity levels. These changes affected some people’s ability to be active more than others (3). For example, people who could access safe, walkable neighborhoods or who worked at home may have increased their physical activity. Understanding prevalence patterns of people who are physically inactive (or who participate in no leisure-time physical activity) before and during the pandemic can provide insight into who initiates any physical activity during large public health emergencies.

The Behavioral Risk Factor Surveillance System (BRFSS) is the only national public health surveillance system that had consistent measures of physical inactivity before and during the pandemic. This study examined changes in prevalence of physical inactivity between 2016, 2018, and 2020, with a focus on changes during 2020 relative to 2018, in the US overall and across sociodemographic groups.

## Methods

Data were from the 2016, 2018, and 2020 BRFSS, a national state-based system of health-related telephone surveys of the civilian, noninstitutionalized US population aged 18 years or older (4). BRFSS data for 50 US states, the District of Columbia, Guam, and Puerto Rico were analyzed. Alternating years of data were analyzed because of annual fluctuation in physical inactivity prevalence, possibly attributable to differences in question order (4,5). Participants were asked, “During the past month, other than your regular job, did you participate in any physical activities or exercises such as running, calisthenics, golf, gardening, or walking for exercise?” Participants who reported no were classified as physically inactive in leisure time (hereinafter, “inactive”), and participants with missing data or who reported “don’t know/not sure” or “refused” (0.2% each year) were excluded. A total of 484,244; 436,741; and 401,276 people were included in analyses for 2016, 2018, and 2020, respectively. The proportion of BRFSS respondents per month ranged from 6.4% to 10.9% (including during 2020).

Participants self-reported their sex, age, race and ethnicity, education, and income. Urban or rural designation of participant residence was based on the 2013 National Center for Health Statistics urban–rural classification scheme for counties (6).

Prevalence differences with 95% CIs were calculated, comparing inactivity between 2020 and 2018 across sociodemographic characteristics overall and stratified by sex. To determine if there were also changes between 2018 and 2016, prevalence differences between these years were calculated. Prevalence differences with 95% CIs that excluded zero were considered statistically significant. Analyses accounted for complex survey design and nonresponse and were conducted using SAS version 9.4 (SAS Institute, Inc) and SUDAAN version 11.0.1 (RTI International). Institutional review board approval was not required because no personal identifiers were included in the data file. The study was conducted according to applicable federal law and Centers for Disease Control and Prevention policy.

## Results

The prevalence of physical inactivity was 24.4%, 24.5%, and 23.8% in 2016, 2018, and 2020, respectively (Figure, Table 1). Inactivity decreased overall by 0.7 percentage points (PP) in 2020 compared with 2018 (95% CI, −1.2 to −0.3). Significant decreases were observed among people aged 45 to 64 years (−1.5 PP [95% CI, −2.3 to −0.7]) and 65 years or older (−1.3 PP [95% CI, −2.1 to −0.4]); women (−1.2 PP [95% CI, −1.8 to −0.6]); people who were non-Hispanic White (−1.5 PP [95% CI, −2.0 to −1.1]); and people living in rural counties (−2.6 PP [95% CI, –3.8 to −1.4]) and, to a lesser extent, urban counties (−0.6 PP [95% CI, –1.0 to −0.1]). Observed changes in 2020 for other groups, including racial and ethnic minority groups, were not statistically significant. No significant decreases observed from 2018 to 2020 were also observed from 2016 to 2018 (Table 1).

**Figure:**
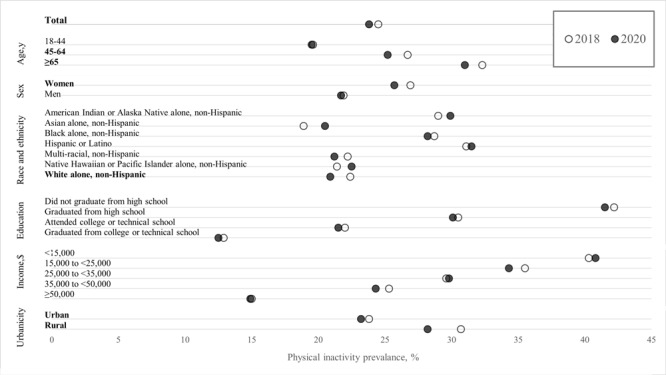
Prevalence of leisure-time physical inactivity, by sociodemographic characteristics, among US adults aged ≥18 years, Behavioral Risk Factor Surveillance System, 2018 and 2020. Prevalence estimates were weighted to account for complex survey design and nonresponse. Bolded groups indicate that changes in prevalence during 2020 compared with 2018 were statistically significant.

**Table 1:** Prevalence of Physical Inactivity Among US Adults Aged ≥18 Years, Behavioral Risk Factor Surveillance System, 2016, 2018, and 2020

Sociodemographic characteristic	Sample size^a^			Prevalence of physical inactivity^b^, % (95% CI)^c^			Prevalence percentage point change (95% CI)^c^	
	2016	2018	2020	2016	2018	2020	2018 vs 2016	2020 vs 2018
**Total**	484,244	436,741	401,276	24.4 (24.2–24.7)	24.5 (24.2–24.8)	23.8 (23.4–24.1)	0.1 (−0.3 to 0.5)	−0.7 (−1.2 to −0.3)^d^
**Age, y**								
18–44	129,089	124,165	121,059	19.2 (18.8–19.6)	19.6 (19.1–20.0)	19.5 (19.0–20.0)	0.3 (−0.2 to 0.9)	−0.1 (−0.7 to 0.6)
45–64	178,642	152,872	134,722	26.9 (26.5–27.4)	26.7 (26.2–27.2)	25.2 (24.6–25.8)	−0.3 (−0.9 to 0.4)	−1.5 (−2.3 to −0.7)^d^
≥65	169,908	151,328	137,326	32.4 (31.9–32.9)	32.3 (31.7–32.8)	31.0 (30.4–31.7)	−0.1 (−0.9 to 0.6)	−1.3 (−2.1 to −0.4)^d^
**Sex**								
Women	274,456	238,587	217,692	26.6 (26.3–27.0)	26.9 (26.5–27.3)	25.7 (25.2–26.2)	0.3 (−0.3 to 0.8)	−1.2 (−1.8 to −0.6)^d^
Men	209,728	197,093	183,584	22.1 (21.7–22.4)	21.9 (21.5–22.3)	21.7 (21.3–22.2)	−0.2 (−0.7 to 0.4)	−0.2 (−0.8 to 0.4)
**Race/ethnicity^e^**								
American Indian or Alaska Native alone, non-Hispanic	7,211	7,533	6,798	26.5 (24.7–28.5)	29.0 (26.4–31.7)	29.9 (27.0–33.0)	2.5 (−0.6 to 5.5)	0.9 (−2.8 to 4.6)
Asian alone, non-Hispanic	10,454	9,893	10,130	19.9 (18.4–21.5)	18.9 (17.4–20.5)	20.5 (18.6–22.5)	−1.0 (−3.2 to 1.3)	1.5 (−1.0 to 4.0)
Black alone, non-Hispanic	38,657	35,886	30,084	29.5 (28.7–30.4)	28.7 (27.9–29.6)	28.2 (27.2–29.2)	−0.8 (−2.0 to 0.4)	−0.5 (−1.8 to 0.8)
Hispanic or Latino	38,997	36,907	36,269	30.9 (30.0–31.7)	31.1 (30.1–32.0)	31.5 (30.4–32.6)	0.2 (−1.1 to 1.5)	0.4 (−1.1 to 1.9)
Multiracial, non-Hispanic	9,406	8,491	8,289	21.6 (20.0–23.4)	22.2 (20.5–24.0)	21.2 (19.2–23.3)	0.5 (−1.9 to 2.9)	−1.0 (−3.6 to 1.7)
Native Hawaiian or Pacific Islander alone, non-Hispanic	1,429	2,087	2,007	18.4 (15.0–22.5)	21.4 (18.1–25.1)	22.5 (17.9–27.9)	3.0 (−1.3 to 7.2)	1.1 (−3.1 to 5.3)
White alone, non-Hispanic	367,359	324,339	295,477	22.1 (21.9–22.4)	22.4 (22.1–22.7)	20.9 (20.6–21.2)	0.3 (−0.1 to 0.7)	−1.5 (−2.0 to −1.1)^d^
**Education level**								
Did not graduate from high school	37,553	32,485	26,163	42.5 (41.6–43.5)	42.2 (41.1–43.4)	41.5 (40.2–42.8)	−0.3 (−1.7 to 1.2)	−0.8 (−2.5 to 1.0)
Graduated from high school	135,956	118,816	106,867	30.1 (29.6–30.6)	30.5 (29.9–31.0)	30.1 (29.4–30.7)	0.4 (−0.3 to 1.2)	−0.4 (−1.2 to 0.5)
Attended college or technical school	132,943	119,814	111,223	21.4 (21.0–21.9)	22.0 (21.5–22.5)	21.5 (21.0–22.1)	0.6 (−0.1 to 1.2)	−0.5 (−1.2 to 0.3)
Graduated from college or technical school	176,082	164,070	155,197	12.5 (12.2–12.8)	12.9 (12.6–13.3)	12.5 (12.1–12.9)	0.4 (0.0 to 0.9)^d^^,^ f	−0.4 (−0.9 to 0.1)
**Income, $**								
<15,000	41,385	34,730	26,539	40.2 (39.2–41.2)	40.3 (39.2–41.5)	40.8 (39.2–42.3)	0.1 (−1.4 to 1.6)	0.5 (−1.5 to 2.4)
15,000 to <25,000	68,496	57,974	48,674	34.3 (33.6–35.1)	35.5 (34.7–36.4)	34.3 (33.3–35.3)	1.2 (0.1 to 2.3)^d^	−1.3 (−2.6 to 0)
25,000 to <35,000	43,861	37,776	31,361	29.3 (28.4–30.2)	29.6 (28.6–30.7)	29.8 (28.6–31.1)	0.4 (−1.0 to 1.7)	0.2 (−1.4 to 1.8)
35,000 to <50,000	58,115	49,503	43,781	24.6 (23.9–25.4)	25.3 (24.5–26.2)	24.3 (23.4–25.2)	0.7 (−0.5 to 1.9)	−1.1 (−2.4 to 0.2)
≥50,000	191,528	180,953	171,167	14.5 (14.2–14.9)	15.0 (14.7–15.4)	14.9 (14.5–15.4)	0.5 (0.0 to 1.0)^d^^,^ f	−0.1 (−0.7 to 0.5)
**Urbanicity^g^**								
Urban	404,189	365,163	335,190	23.8 (23.5–24.1)	23.8 (23.5–24.1)	23.2 (22.9–23.5)	0.0 (−0.4 to 0.4)	−0.6 (−1.0 to −0.1)^d^
Rural	72,687	65,096	58,963	30.3 (29.5–31.1)	30.7 (29.9–31.6)	28.2 (27.3–29.0)	0.5 (−0.7 to 1.7)	−2.6 (−3.8 to −1.4)^d^

a Among people with data on physical activity, missingness in sociodemographic characteristics varied from 16.7%–19.9% for income to <3% for sex, age, race and ethnicity, education, and urban/rural status across 2016, 2018, and 2020. Values for n for demographic groups may not sum to the total N (484,244 for 2016; 436,741 for 2018; and 401,276 for 2020) due to missing demographic information; those with missing demographic information were excluded from the analytic sample only for the analyses of that demographic characteristic.

b Participants were asked, “During the past month, other than your regular job, did you participate in any physical activities or exercises such as running, calisthenics, golf, gardening, or walking for exercise?” Participants who reported no were classified as physically inactive.

c All estimates were weighted to account for complex survey design and nonresponse.

d Indicates that the 95% CI excludes zero and the difference in prevalence is therefore statistically significant.

e People of another race were included in analyses; however, estimates are not shown for this group due to the heterogeneity of the category.

f Lower CI is 0.01 and rounds to zero.

g Urbanicity is based on the 2013 National Center for Health Statistics urban–rural classification scheme for counties (6).

Inactivity significantly declined among multiple subgroups of women in 2020 versus 2018 (Table 2). The largest decreases occurred among rural-dwelling women (−3.1 PP [95% CI, –4.7 to −1.5]), followed by White women (−2.1 PP [95% CI, –2.7 to –1.5]). Significant decreases were also observed among women aged 45 to 64 years (−1.6 PP [95% CI, –2.7 to −0.5]) and aged 65 years or older (−1.6 PP [95% CI, –2.7 to −0.4]), women who graduated from high school (−1.9 PP [95% CI, –3.2 to −0.7]) or from college or technical school (−0.9 PP [95% CI, –1.6 to −0.2]), women making $35,000 to less than $50,000 per year (−1.8 PP [95% CI, –3.6 to −0.03]), and women living in urban counties (−1.1 PP [95% CI, –1.7 to −0.4]). In 2020, inactivity significantly declined among 3 subgroups of men: rural-dwelling men (−2.1 PP [95% CI, –3.8 to −0.3]), men aged 45–64 years (−1.4 PP [95% CI, –2.5 to −0.3]), and White men (−0.9 PP [95% CI, –1.5 to −0.3). No significant sex-specific decreases observed from 2018 to 2020 were also observed from 2016 to 2018.

**Table 2:** Changes in Prevalence of Physical Inactivity, by Sociodemographic Group and Sex, Among US Adults Aged ≥18 Years, Behavioral Risk Factor Surveillance System, 2016, 2018, and 2020

Characteristic	Women		Men	
	Prevalence percentage point change (95% CI)^a^		Prevalence percentage point change (95% CI)^a^	
	2018 vs 2016	2020 vs 2018	2018 vs 2016	2020 vs 2018
**Total**	0.3 (−0.3 to 0.8)	−1.2 (−1.8 to −0.6)^b^	−0.2 (−0.7 to 0.4)	−0.2 (−0.8 to 0.4)
**Age, y**				
18–44	0.7 (−0.2 to 1.5)	−0.8 (−1.8 to 0.1)	0.0 (−0.8 to 0.8)	0.7 (−0.2 to 1.6)
45–64	0.1 (−0.8 to 1.1)	−1.6 (−2.7 to −0.5)^b^	−0.7 (−1.7 to 0.2)	−1.4 (−2.5 to −0.3)^b^
≥65	−0.5 (−1.6 to 0.5)	−1.6 (−2.7 to −0.4)^b^	0.4 (−0.8 to 1.5)	−0.8 (−2.1 to 0.5)
**Race/ethnicity**				
American Indian or Alaska Native, non-Hispanic	4.9 (−0.6 to 9.2)	−1.1 (−5.7 to 3.4)	0.0 (−3.7 to 3.7)	2.9 (−2.2 to 7.9)
Asian, non-Hispanic	0.1 (−3.3 to 3.5)	1.7 (−2.2 to 5.6)	−2.1 (−4.8 to 0.6)	1.6 (−1.6 to 4.8)
Black, non-Hispanic	−0.3 (−1.9 to 1.3)	−0.7 (−2.5 to 1.1)	−1.4 (−3.2 to 0.4)	−0.3 (−2.2 to 1.5)
Hispanic or Latino	0.2 (−1.6 to 2.0)	−0.1 (−2.3 to 2.0)	0.2 (−1.6 to 2.1)	1.0 (−1.1 to 3.1)
Multiracial, non-Hispanic	−0.9 (−4.4 to 2.6)	−0.8 (−4.8 to 3.3)	1.5 (−1.5 to 4.6)	−1.2 (−4.4 to 2.0)
Native Hawaiian or Pacific Islander, non-Hispanic	0.9 (−4.3 to 6.1)	2.9 (−2.3 to 8.1)	4.3 (−0.8 to 9.4)	0.2 (−5.4 to 5.8)
White, non-Hispanic	0.5 (−0.1 to 1.0)	−2.1 (−2.7 to −1.5)^b^	0.0 (−0.6 to 0.5)	−0.9 (−1.5 to −0.3)^b^
**Education level**				
Did not graduate from high school	−0.8 (−2.8 to 1.2)	0.3 (−2.2 to 2.8)	0.2 (−1.8 to 2.3)	−1.8 (−4.2 to 0.5)
Graduated from high school	1.4 (0.4 to 2.5)^b^	−1.9 (−3.2 to −0.7)^b^	−0.4 (−1.4 to 0.5)	1.1 (0.0 to 2.2)^c^
Attended college or technical school	0.6 (−0.3 to 1.5)	−0.8 (−1.8 to 0.2)	0.5 (−0.5 to 1.4)	0.0 (−1.1 to 1.0)
Graduated from college or technical school	0.8 (0.2 to 1.5)^b^	−0.9 (−1.6 to −0.2)^b^	−0.1 (−0.6 to 0.5)	0.1 (−0.5 to 0.8)
**Income level, $**				
<15,000	0.8 (−1.2 to 2.8)	−0.2 (−2.6 to 2.3)	−1.0 (−3.3 to 1.3)	1.3 (−1.7 to 4.3)
15,000 to <25,000	1.0 (−0.5 to 2.5)	−1.3 (−3.0 to 0.5)	1.3 (−0.4 to 2.9)	−1.1 (−3.0 to 0.8)
25,000 to <35,000	0.3 (−1.6 to 2.1)	0.3 (−1.8 to 2.4)	0.5 (−1.6 to 2.6)	0.0 (−2.3 to 2.4)
35,000 to <50,000	1.0 (−0.6 to 2.6)	−1.8 (−3.6 to 0.0)^b^^,^ d	0.4 (−1.3 to 2.1)	−0.3 (−2.2 to 1.6)
≥50,000	1.0 (0.3 to 1.7)^b^	−0.3 (−1.2 to 0.6)	0.1 (−0.6 to 0.7)	0.0 (−0.7 to 0.8)
**Urbanicity**				
Urban	0.2 (−0.4 to 0.8)	−1.1 (−1.7 to −0.4)^b^	−0.3 (−0.8 to 0.3)	−0.1 (−0.7 to 0.6)
Rural	0.5 (−1.1 to 2.1)	−3.1 (−4.7 to −1.5)^b^	0.5 (−1.2 to 2.3)	−2.1 (−3.8 to −0.3)^b^

a Prevalence estimates and differences were weighted to account for complex survey design and nonresponse. Prevalence differences with 95% CIs not containing zero were considered statistically significant. People of another race were included in analyses; however, estimates are not shown for this group due to the heterogeneity of the category.

b Indicates that the 95% CI excludes zero and the difference in prevalence is therefore significant.

c Lower CI is −0.04 and rounds to zero.

d Upper CI is −0.03 and rounds to zero.

## Discussion

Leisure-time physical inactivity modestly decreased overall and among specific US subpopulations in 2020 compared with 2018. Overall, the observed 0.7 percentage point decrease in physical inactivity suggests that nearly 1.8 million fewer US adults in 2020 were physically inactive during leisure time, and avoiding inactivity is a key recommendation for adults in the second edition of the *Physical Activity Guidelines for Americans* (1).

Prior surveys (3,7–9) on physical activity changes during the pandemic produced discrepant findings, which may be due to varied methodologies (eg, device- vs questionnaire-based assessment) or a focus on different domains of activity (7). The current study describes changes in the prevalence of people participating in *no* leisure-time physical activity. While this study does not measure changes in quantified levels of activity, initiating any activity is an important first step given relatively stagnant levels of physical inactivity before the pandemic (5).

Studies have documented persistent disparities in physical activity across racial and ethnic and socioeconomic groups (3,9), with potential widening of disparities during the pandemic (8). The 2020 decrease in leisure-time physical inactivity (or the increase in initiation of any leisure-time physical activity) we found among some populations, but not others, may result from different physical activity opportunities and access to safe spaces (9,10) across subpopulations during the pandemic. Additional research of structural determinants, such as occupational requirements (eg, remote work) affecting availability for leisure-time physical activity, may also help to explain differential decreases. Less traffic, which is more commonly reported as a barrier to walking among rural compared with urban residents (11), or access to new or changed spaces (12), may have also helped some populations be more active. Additionally, some groups experiencing disproportionate health impacts early in the pandemic (eg, people from racial and ethnic minority groups) may have had concerns over COVID-19 exposure during some physical activities (3,13), which may partially explain the decrease in inactivity among people who are White but not people from racial and ethnic minority groups.

This study has limitations. First, data on physical activity were limited to nonoccupational, leisure-time activity. Second, data were self-reported and may be subject to social desirability and other recall biases. Third, patterns of physical inactivity may have differed across periods of 2020. Fourth, this study did not identify causal factors (eg, policies) related to the pandemic that influenced patterns of physical activity. Finally, analyses did not control for multiple comparisons, and sample sizes for some groups limited the ability to statistically detect changes.

This study highlights a need to understand and address factors influencing differential changes in leisure-time physical inactivity across subpopulations during public health emergencies.
